# Solving singularly perturbed fredholm integro-differential equation using exact finite difference method

**DOI:** 10.1186/s13104-023-06488-8

**Published:** 2023-09-28

**Authors:** Solomon Regasa Badeye, Mesfin Mekuria Woldaregay, Tekle Gemechu Dinka

**Affiliations:** Department of Applied Mathematics, Adama Science and Technology University, Adama, Ethiopia

**Keywords:** Primary 65L10, Secondary 65D30, 65L20, 65L70

## Abstract

**Objectives:**

In this paper, a numerical scheme is designed for solving singularly perturbed Fredholm integro-differential equation. The scheme is constructed via the exact (non-standard) finite difference method to approximate the differential part and the composite Simpson’s 1/3 rule for the integral part of the equation.

**Result:**

The stability and uniform convergence analysis are demonstrated using solution bound and the truncation error bound. For three model examples, the maximum absolute error and the rate of convergence for different values of the perturbation parameter and mesh size are tabulated. The computational result shows, the proposed method is second-order uniformly convergent which is in a right agreement with the theoretical result.

## Introduction

Numerous phenomenons in science and engineering are characterized by a rapid transition of the observable quantity, such as shock waves in fluid movements, boundary layer flow along the floor of a body, and edge results in elastic plate deformation. The mathematical models describing these phenomena incorporate a small parameter(s), and the effect of these parameter(s) displays a sudden alternate of the dependent variable, taking place within a small region. The solution of the boundary layer flow problem and that of the elastic plate are characterized by the reality that the small perturbation has an observable impact solely in the vicinity of the boundary, and therefore, one uses the term “singular perturbations of boundary layer type” [14].

Singularly perturbed differential equations are typically characterized by a small parameter ($\varepsilon$) multiplying some or all of the highest-order derivative terms in the differential equation. In general, the solutions to such equations exhibit multi-scale phenomena [20]. Within certain thin sub-regions of the domain, the scale of some derivatives is significantly larger than other derivatives [10, 27]. These thin regions of rapid change are referred to as boundary layers [1].

Many mathematical formulations in natural science, i.e., the study of fluids, biology, and chemical kinetics, contain integro-differential equations [5, 21, 23, 24]. It can be classified into two types, i.e., Fredholm and Volterra equations. Volterra equations have the upper bound limit as a variable, while the Fredholm equation has a fixed bound of limits. Numerous works on the numerical treatment of Fredholm/Volterra integral equations have been developed. To list a few of them: In [6] a fast multiscale Galerkin method is developed. In [13] several numerical approaches are proposed for the solution of Fredholm integro-differential equations modelling neural networks. The solution strategy is to use expansions onto standard cardinal basis functions and the collocation method. The Adomian decomposition method is used in [15], an iterative method based on the least square QR factorization method is used in [16], a comparison between the variational iteration method and trapezoidal rule is discussed in [25].

In this paper, we focus on singularly perturbed Fredholm integro-differential equations. Singularly perturbed integral equations or integro-differential equations are shown in the mathematical model of population variability, polymer rhythm, and glucose tolerance [7]. In particular, the singularly perturbed Fredholm integral equation is given by optimal control problems [22]. It is known that, unless strong constraints are made on the step size of a discretization, most of the classical numerical methods are not fit to handle the problems with a small parameter multiplying the derivative. The truncation error becomes unbounded as the perturbation parameter gets small. Due to this, the numerical treatment of singularly perturbed problems presents severe difficulties that have to be addressed to ensure accurate numerical solutions [4]. As a result, in recent years, few works on the numerical solution of singularly perturbed Fredholm/Volterra integral equations have been recorded in the literature [11, 12]. Durmaz et al. [8] developed a fitted difference scheme on Shishkin mesh using interpolating quadrature rules and an exponential basis function for the numerical treatment of the singularly perturbed Fredholm integro-differential equation with mixed boundary conditions.

To solve the initial-value problem for a singularly perturbed Fredholm integro-differential equation, Amiraliyev et al. [2] proposed a fitted finite difference scheme on a uniform mesh. The difference scheme was via the method of integral identities with the use of exponential basis functions and interpolating quadrature rules with the weight. The method exhibits a first-order uniform convergence. Amiraliyev et al. in [1] applied a fitted mesh method for solving the singularly perturbed Volterra delay-integro-differential equation. The difference scheme was constructed based on the method of integral identity by using interpolating quadrature rules.

Kudu et al. in [17] constructed a numerical method for first-order singularly perturbed delay integro-differential equations. They used implicit difference rules to discretize the differential part and composite quadrature rules for the integral part. The authors in [7] presented a difference scheme to solve singularly perturbed Fredholm integro-differential equations. The difference scheme was constructed via the method of integral identities using interpolating quadrature rules with remainder terms in integral form. Their method is first-order convergent. Amiraliyev et al. [3] proposed a fitted finite difference scheme on the uniform mesh to solve the problem in (1). The difference scheme was constructed via the method of integral identities with the use of exponential basis functions and interpolating quadrature rules with weight and remainder terms in integral form. They discussed the convergence of the method and showed that it has first-order convergence. On the other hand, Durmaz et al. [9] proposed an exponentially fitted difference scheme on Shishkin mesh for the numerical solution of the problem in (1). The fitting factor was introduced via the method of integral identities with the use of exponential basis functions and interpolating quadrature rules with weight and remainder terms in integral form.

The objective of this paper is to develop an accurate and uniformly convergent numerical method for solving singularly perturbed Fredholm integro-differential equation. We used an exact (non-standard) finite difference method together with a composite Simpson’s 1/3 rule for approximating the problem; we established the stability analysis and the uniform convergence of the scheme.

### Notation 1.1

The parameter *C* in this paper is a generic positive constant that is independent of the perturbation parameter $\varepsilon$ and the mesh parameter $h = \frac{1}{N}$. The norm $\Vert .\Vert$ used in this paper is the maximum norm which is defined as $\Vert g\Vert =\max _{x\in [0, l]}|g(x)|$.

## Problem statement

We considered a singularly perturbed Fredholm integro-differential equation (SPFIDE) of the form:

1 $$\begin{aligned} \begin{aligned} L_{\varepsilon }u:=- \varepsilon u''(x) + a(x)u(x) +\lambda \int _{0}^{l}K(x,s)u(s)ds=f(x),  x \in (0,l),\\ u(0)=A,  u(l)=B, \end{aligned} \end{aligned}$$

where $\varepsilon \in (0,1]$ is the perturbation parameter and $\lambda$, *A*, *B* are given constant. It ia assumed that $a(x)\ge \alpha > 0$, *f*(*x*) and kernel function *K*(*x*, *s*) are the sufficiently smooth functions satisfying certain regularity conditions to be specified. Under these conditions, the solution *u*(*x*) of (1) exhibits a boundary layer at $x = 0$ and $x = l$ [3].

### Properties of the continuous solution

In this subsection, we analyse some properties of the continuous solution (1) which guarantee the existence and uniqueness of the exact solution. A replication of this property in the discrete form is used to analyze the numerical method which is presented in “Numerical discretization” section.

#### Lemma 2.1

[9, 17] (The maximum principle) Let $u \in C^2[0,l] \cap C^0[0,l],$ and

$$ |\lambda | < \frac{\alpha }{{\max _{{0 \le x \le l}} \int_{0}^{l} | K(x,s)|ds}}, $$

with $u(0)\ge 0, u(l)\ge 0$ and $L_{\varepsilon }u(x)\ge 0$ for $x \in (0,l)$ then it holds that $u(x)\ge 0, \; x\in [0,l]$.

#### Lemma 2.2

[9, 17] If $$ a, f \in C^2[0,l], \frac{\partial ^sK}{\partial x^s}\in C[0,l]^2 $$, ($$s=0,1,2 $$) then the solution *u*(*x*) of (1) hold the following bounds

2 $$\begin{aligned} \Vert u\Vert \le C \end{aligned}$$

3 $$\begin{aligned} |u^{(k)}(x)|\le C\left\{ 1+\varepsilon ^{\frac{-k}{2}}\big (e^{-\frac{\sqrt{\alpha }x}{\sqrt{\varepsilon }}}+e^{-\frac{\sqrt{\alpha }(1-x)}{\sqrt{\varepsilon }}}\big )\right\} . \end{aligned}$$

#### Definition 2.1

A numerical scheme (or method) is said to be exact method, if the differential equation has the same solution as the difference scheme at the grid point $x_{i}$ (i.e. there is no discretization error at the grid points).

## Numerical discretization

In this section, we used the exact (non-standard) finite-difference method together with composite Simpson’s 1/3 rule to discretize the SPFIDE. The differential part will be approximated by using the exact finite-difference method. In order to construct exact finite difference method we follow the Mickens rules in [19]. Consider the constant coefficient sub-equations given by:

4 $$\begin{aligned} - \varepsilon u''(x) + \alpha u(x) =0, \end{aligned}$$

where $a(x) \ge \alpha > 0$. Thus, the equation in (4) has two linearly independent solutions namely $e^{(\lambda _{1} x)}$ and $e^{(\lambda _{2}x)}$ with $\lambda _{1,2} = \pm \sqrt{\frac{\alpha }{\varepsilon }}.$

On the domain [0, *l*] , using uniform mesh with mesh length $\Delta x=h$ such that $\Omega _{N} = \left\{ x_{i}= ih, i= 1, 2,..., N, \ x_{0}=0, \ x_{N}=l, \ h= \frac{l}{N}\right\}$ where *N* is the number of mesh points. Let us denote the approximate solution of *u*(*x*) at $x_{i}$ by $U_{i}$. The objective is to calculate a difference equation which has the same general solution as the differential equation in (4) at the grid point $x_{i}$ given by $u_{i} = A_{1} e^{(\lambda _{1} x_{i} )} + A_{2} e^{(\lambda _{2} x_{i} )}$. Using the theory of difference equations for second order linear difference equations in [19], we have

5 $$ \left| {\begin{array}{*{20}c}    {u_{{i - 1}} \,e^{{\lambda _{1} x_{{i - 1}} }} \,e^{{\lambda _{2} x_{{i - 1}} }} }  \\    {u_{i} \,e^{{\lambda _{1} x_{i} }} \,e^{{\lambda _{2} x_{i} }} }  \\    {u_{{i + 1}} \,e^{{\lambda _{1} x_{{i + 1}} }} \,e^{{\lambda _{2} x_{{i + 1}} }} }  \\   \end{array} } \right| = 0, $$

substituting the values of $\lambda _{1}$and $\lambda _{2}$ and dividing both sides by $e^{\sqrt{\frac{\alpha }{\varepsilon }}h}-e^{-\sqrt{\frac{\alpha }{\varepsilon }}h}$, we obtain:

6 $$\begin{aligned} u_{i-1}-2cosh\left( \sqrt{\frac{\alpha }{\varepsilon }}h\right) u_i+u_{i+1}=0 \end{aligned}$$

which is an exact difference scheme for (4). After doing the arithmetic manipulation and rearrangement on (6), we obtain:

$$\begin{aligned} - \ \varepsilon \frac{u_{i-1} - \ 2u_{i} + \ u_{i+1}}{\frac{4}{\gamma ^{2}}\sinh ^{2}(\gamma \frac{h}{2})} + \ \alpha u_{i} = 0, \ \gamma =\ \sqrt{\frac{\alpha }{\varepsilon }}. \end{aligned}$$

The denominator function for the discretization of second order derivative is $\psi ^{2} = \ {\frac{4}{\gamma ^{2}}\sinh ^{2}(\gamma \frac{h}{2})}$. Adopting this denominator function for the variable coefficient problem, we can write as:

7 $$\begin{aligned} \psi _{i}^{2}= \frac{4}{\sqrt{\frac{a(x_{i})}{\varepsilon }}^{2}}\sinh ^{2}\left( \sqrt{\frac{a(x_{i})}{\varepsilon }} \frac{h}{2}\right) . \end{aligned}$$

Using the denominator function $\psi _{i}^{2}$ into the main discrete scheme, we obtain the difference scheme as:

8 $$\begin{aligned} \begin{aligned} L_{\varepsilon }^Nu_i\equiv - \varepsilon \frac{u_{i-1} - \ 2u_{i} + \ u_{i+1}}{\psi _{i}^{2}} + \ a_{i} u_{i} + \lambda \int _{0}^{l}K_i(s)u(s)ds =f_i + R_{1}, \end{aligned} \end{aligned}$$

Now, the truncation error of scheme (8) is given by

$$\begin{aligned} \begin{aligned} L_{\varepsilon }^N(u_i-u(x_i))=f_i-L_{\varepsilon }^Nu_i\\=\left( -\varepsilon u^{''}_{i}+a_iu_i+\lambda \int _{0}^{l}K_i(s)u(s)ds\right) \\\quad -\left( - \varepsilon \frac{u_{i-1} - \ 2u_{i} + \ u_{i+1}}{\psi _{i}^{2}} + \ a_{i} u_{i} + \lambda \int _{0}^{l}K_i(s)u(s)ds\right) ,\\=-\varepsilon u^{''}_{i}+\varepsilon \frac{u_{i-1} - \ 2u_{i} + u_{i+1}}{\psi _{i}^2}. \end{aligned} \end{aligned}$$

Taylor series expansions of the terms $u_{i+1}$ and $u_{i-1}$ are given as follows:

$$\begin{aligned} u_{i\pm 1}= u_i\pm hu_i'+\frac{h^2}{2!}u_i''\pm \frac{h^3}{3!}u_i'''+ \frac{h^4}{4!}u_i^{(iv)}\pm \frac{h^5}{5!}u_i^{(v)}+\dots \end{aligned}$$

Using the truncated Taylor series expansions of the terms $u_{i+1}$ and $u_{i-1}$ yields

$$\begin{aligned} L_{\varepsilon }^N(u_i-u(x_i))=-\varepsilon u_i''+\frac{\varepsilon }{\psi _{i}^2}\left( h^2u_i''+\frac{h^4}{12}u_i^{(iv)}(\xi )\right) =R_1, \ \xi \in (x_{i-1}, x_{i+1}). \end{aligned}$$

Moreover applying the composite Simpson 1/3 rule to the integral term in (8). In order to drive composite Simpson’s 1/3 rule substitute $n=2$ in Newton-Cotes quadrature formulae in [26] obtain the following result

$$\begin{aligned} \int _{s_0}^{s_2}K(x_i,s)u(s)ds=\frac{h}{3}\left[ K(x_i,s_0)u(s_0)+4K(x_i,s_1)u(s_1)+K(x_i,s_2)\right] , \end{aligned}$$

where $K(x_i,s_j)=K_{ij}\ u(s_j)=u_j$ for $j=0,\ 1,\dots ,\ n$. Now, the error of this method can be approximated in the following way.

Let the function $y=K(x_i,s)u(s)$ be continuous and possess a continuous derivatives in $[s_0,s_2]$. Expanding *y* about $s=s_0$ we obtain

9 $$\begin{aligned} y(x) & =y_0+(s-s_0)y_0'+\frac{1}{2}(s-s_0)^2y_0''+\frac{1}{3!}(s-s_0)^3y_0'''+\frac{1}{4!}(s-s_0)^4y_0^{(iv)}+\dots  \\ & \int _{s_0}^{s_2}K(x_i,s)u(s)ds=\int _{0}^{2}h\left( y_0+phy_0'+\frac{(ph)^2}{2}y_0''+\frac{(ph)^3}{3!}y_0'''+\frac{(ph)^4}{4!}y_0^{(iv)}+\dots \right) dp  \\ & \quad =h\left[ py_0+\frac{p^2h}{2}y_0'+\frac{(ph)^2}{6}py_0''+\frac{(ph)^3}{24}py_0'''+\frac{(ph)^4}{120}py_0^{(iv)}+\dots \right] _0^2,\nonumber \\ & \quad =2hy_0+2h^2y_0'+\frac{4h^3}{3}y_0''+\frac{2h^4}{3}y_0'''+\frac{4h^5}{15}y_0^{(iv)}+\dots . \end{aligned}$$

Therefore

10 $$\begin{aligned}{}  {} y_0=y_0, \end{aligned}$$

11 $$\begin{aligned}{}  {} y_1=y_0+hy_0'+\frac{h^2}{2}y_0''+\frac{h^3}{6}y_0'''+\frac{h^4}{24}y_0^{(iv)}+\dots , \end{aligned}$$

12 $$\begin{aligned}{}  {} y_2=y_0+2hy_0'+2h^2y_0''+\frac{4h^3}{3}y_0'''+\frac{2h^4}{3}y_0^{(iv)}+\dots . \end{aligned}$$

From (10), (11) and (12), we get:

13 $$\begin{aligned}  \frac{h}{3}\left[ y_0+4y_1+y_2\right]=\frac{h}{3}\left[ 6y_0+6hy_0'+4h^2y_0''+2h^3y_0'''+\frac{5h^4}{6}y_0^{(iv)}+\dots ,\right] \\=2hy_0+2h^2y_0'+\frac{4h^3}{3}y_0''+\frac{2h^4}{3}y_0'''+\frac{5h^5}{18}y_0^{(iv)}+\dots .  \end{aligned}$$

From (9) and (13) we obtain,

$$\begin{aligned} \int _{s_0}^{s_2}yds-\frac{h}{3}\left[ y_0+4y_1+y_2\right] =\frac{-1}{90}h^5y_0^{(iv)}. \end{aligned}$$

This is the error committed in the interval $[s_0, s_2]$.

Generally, the composite Simpson’s 1/3 rule needs an even number of sub-divisions. Let [0, *l*] be sub-divided into *N* even number of sub-divisions, $0=s_0<s_1<s_2<\dots <s_N=l$, the integral over the whole interval is found by adding these integrations and is equal to

$$\begin{aligned} \int _{s_0}^{s_N}yds=\frac{h}{3}\left( y_0+4\sum _{j=1}^{N/2}y_{2j-1}+2\sum _{j=1}^{(N/2)-1}y_{2j}+y_N\right) . \end{aligned}$$

We obtain the errors in the intervals [0, *l*] as

$$\begin{aligned} R_2=\frac{-1}{90}h^5\left[ y_0^{(iv)}+y_2^{(iv)}+\dots +y_{N-2}^{(iv)}\right] =\frac{-l}{180}h^4u^{(iv)}(\xi ),\ \xi \in [0,l], \end{aligned}$$

where $u^{(iv)}(\xi )$ is the largest value of the *N*-quantities on 4th derivatives. Therefore, the integral term in (8) is approximated as:

14 $$\begin{aligned} \begin{aligned} \int _{0}^{l}K(x_i,s)u(s)ds=\,\frac{h}{3}\left( 4\sum _{j=1}^{N/2}K(x_i,s_{2j-1})u(s_{2j-1})+2\sum _{j=1}^{N/2-1}K(x_i,s_{2j})u(s_{2j})\right) \\\quad +\frac{h}{3}\left( K(x_i,s_0)u(s_0)+K(x_i,s_N)u(s_N)\right) +R_2. \end{aligned} \end{aligned}$$

From (8) and (14) for $i=1,2, \dots N-1,$ we have the following relation

15 $$\begin{aligned} \begin{aligned} L_\varepsilon ^Nu_i:= - \varepsilon \frac{u_{i-1} - \ 2u_{i} + \ u_{i+1}}{\psi _{i}^{2}} + \ a_{i} u_{i} +\lambda h\sum _{j=0}^{N}\eta _jK_{ij}u_j=f_i-R, \end{aligned} \end{aligned}$$

where $$ R=-R_1-R_2 $$ and

$$ \eta _{j}  = \left\{ \begin{gathered}   \frac{1}{3},\,{\text{for}}\;j = 0,N, \hfill \\   \frac{4}{3},\,{\text{for}}\;j = 1,3,5, \ldots ,N - 1, \hfill \\   \frac{2}{3},\,{\text{for}}\;j = 2,4,6, \ldots ,N - 2. \hfill \\  \end{gathered}  \right.  $$

Based on (15) we propose the following difference scheme for approximating (1).

16 $$\begin{aligned} \begin{aligned} L_\varepsilon ^Nu_i=- \varepsilon \frac{u_{i-1} - 2u_{i} + u_{i+1}}{\psi _{i}^{2}} + a_{i} u_{i} +\lambda h\sum _{j=0}^{N}\eta _jK_{ij}u_j=f_i, \ i=1,2, \dots N-1, \\ u_0=A,  u_N=B. \end{aligned} \end{aligned}$$

Lastly, from (16) the linear system equations for $u_1, u_2, u_3, \dots , u_{N-1}$ are generated. Therefore, the generated system of linear algebraic equations can be written in matrix form of

17 $$\begin{aligned} (M + S)u=F, \end{aligned}$$

where *M* and *S* are coefficient matrix, *F* is a given function and *u* is an unknown function which is to be determined. The entries of *M*, *S* and *F* are given as:

$$\begin{aligned} M = \left\{ \begin{array}{ll} a_{ii}= \frac{2 \varepsilon }{\psi _{i}^{2}} +a(x_{i}), {} \text{ for } i=1, 2, \dots , N-1, \\ a_{ii+1}= \frac{- \varepsilon }{\psi _{i}^{2}}, {} \text{ for } i= 1, 2, \dots , N-2, \\ a_{ii-1}= \frac{- \varepsilon }{\psi _{i}^{2}}, {} \text{ for } i= 2, 3, \dots , N-1, \end{array} \right. \end{aligned}$$

$$\begin{aligned} S=\left\{ \begin{array}{ll}\frac{4\lambda h}{3}K_{i,2j-1},  j= 1, 2, \dots , \frac{N}{2},\\ \frac{2\lambda h}{3}K_{i,2j},  j= 1, 2, \dots , \frac{N}{2}-1, \end{array} \right. \end{aligned}$$

and

$$\begin{aligned} F= \left\{ \begin{array}{l} f_{1} -\left( (\lambda h\eta _0K_{1,0}-\frac{\varepsilon }{\psi _{1}^2})A+\lambda h\eta _NK_{1,N}B\right) ,\\ f_{i}-\lambda h\left( \eta _0K_{i,0}A+\eta _NK_{i,N}B\right) , for\ i= 2, 3, \dots , N-2, \\ f_{N-1} - \left( \lambda h\eta _0K_{N-1,0}A +(\lambda h\eta _NK_{N-1,N}-\frac{ \varepsilon }{\psi _{N}^{2}})B\right) . \end{array} \right. \end{aligned}$$

## Stability and convergence analysis

In this section, we need to show the discrete scheme in (16) satisfy the discrete maximum principle, uniform stability estimates, and uniform convergence. The difference operator, $L_{\varepsilon }^{N}$ satisfies the the following lemma.

### Lemma 4.1

(Discrete maximum principle) Assume that the mesh function $\Psi _{i}$ satisfies $\Psi _{0} \ge 0$ and $\Psi _{N} \ge 0$.Then $L_{\varepsilon }^{N} \varPsi _{i} \ge 0$, $i=1,2 \dots , N-1$, implies that $\Psi _{i} \ge 0$, $\forall i=0,1, \dots , N$.

### Proof

Let *k* be such that $\Psi _{k} = min \Psi _{i}$ and suppose that $\Psi _{k} < 0$. Evidently, $k \notin \left\{ 0,N \right\}$, $\Psi _{k} \le \Psi _{k+1}$ and $\Psi _{k} \le \Psi _{k-1}$. If $\psi _{i}^{2}= \ {\frac{4}{\sqrt{\frac{a(x_{i})}{\varepsilon }}^{2}}\sinh ^{2}(\sqrt{\frac{a(x_{i})}{\varepsilon }} \frac{h}{2})}$, it follows that

$$\begin{aligned} L_{\varepsilon }^{N}\Psi _{k}= -\varepsilon \delta ^{2}\Psi _{k}+a_{k}\Psi _{k}+\lambda h\left( \sum _{j=0}^{N}\eta _jK_{ij}\right) \Psi _{k},\\= -\varepsilon \frac{\varPsi _{k+1}-2\Psi _{k}+\Psi _{k-1}}{\psi _{i}^{2}} + a_{k}\Psi _{k}+\lambda h\left( \sum _{j=0}^{N}\eta _jK_{ij}\right) \Psi _{k},\\= -\varepsilon \frac{(\varPsi _{k+1}-\Psi _{k})+(\Psi _{k-1}- \Psi _{k})}{\psi _{i}^{2}} + a_{k}\Psi _{k}+\lambda h\left( \sum _{j=0}^{N}\eta _jK_{ij}\right) \varPsi _{k}  0, \end{aligned}$$

which is a contradiction. It follows that $\Psi _{k} \ge 0$, and thus that $\Psi _{i} \ge 0, \; \forall i=0,1, \dots , N$. $\square$

The uniqueness of the solution is guaranteed by this discrete maximum principle. The existence follows easily since, as for linear problems, the existence of the solution is implied by its uniqueness [12]. The discrete maximum principle enables us to prove the next lemma which provides the boundedness of the solution.

### Lemma 4.2

If $u_i$ is the solution of the discrete problem (16) then it admits the bound

$$\begin{aligned} |u_i|\le \alpha ^{-1} \max _{x_i\in [0, l]}|L_\varepsilon ^Nu_i|+max\left\{ |A|, |B|\right\} . \end{aligned}$$

### Proof

We consider the functions $\varPsi ^{\pm }$ defined by $\varPsi _{i}^{\pm }= p\pm u_i,$ where $p=\alpha ^{-1} \max _{x_i\in [0, l]}|L_\varepsilon ^Nu_i|+max\left\{ |A|, |B|\right\} .$ At the boundaries we have

$$\begin{aligned} \varPsi _0^{\pm }=p\pm u_0=p\pm A\ge 0, \;\; \varPsi _N^{\pm }=p\pm u_N=p\pm B\ge 0. \end{aligned}$$

Now for $\Omega _{N}$ we have

$$\begin{aligned} L_\varepsilon ^N\varPsi _{i}^{\pm }=-\varepsilon \left( \frac{p\pm u_{i+1}-2(p\pm u_i)+p\pm u_{i-1}}{\psi _{i}^2}\right) +a_i(p\pm u_i)+\lambda \left( p\pm h\sum _{j=0}^{N}\eta _jK_{ij}y_j\right) \\=a_i p\pm L_\varepsilon ^Nu_i,\\=a_i\left( \alpha ^{-1} \max _{x_i\in [0, l]}|L_\varepsilon ^Nu_i|+max(|A|, |B|)\right) \pm f_i \ge 0, \ \text {since} \ a_i\ge \alpha . \end{aligned}$$

From Lemma 3.2 it follows that $\varPsi _{i}^{\pm }\ge 0, \ \forall x_i\in [0,l]$, this completes the proof. $\square$

### Lemma 4.3

[18] For all integers k on a fixed mesh, we have that

$$\begin{aligned} \lim _{\varepsilon \rightarrow 0}\max _{1<i<N-1} \frac{exp(-Cx_{i}/\sqrt{\varepsilon })}{\varepsilon ^{k/2}} = 0 \end{aligned}$$

and

$$\begin{aligned} \lim _{\varepsilon \rightarrow 0}\max _{1<i<N-1} \frac{exp(-C(1-x_{i})/\sqrt{\varepsilon })}{\varepsilon ^{k/2}} = 0, \end{aligned}$$

where $$x_{i} = ih, h = 1/N, i = 1, 2, \dots , N-1$$.

Next, we analyze the uniform convergence of the method. From (15) and (16) for the error of the approximate solution $z_i=u_i-u(x_i)$ we have

18 $$ L_{\varepsilon }^{N} z_{i} : =  - \varepsilon \frac{{z_{{i - 1}}  - \;2z_{i}  + \;z_{{i + 1}} }}{{\psi _{i}^{2} }} + \;a_{i} z_{i}  + \lambda h\sum\limits_{{j = 0}}^{N} {\eta _{j} } K_{{ij}} z_{j} ,\;i = 1,2, \ldots ,N - 1,\,z_{0}  = 0,z_{N}  = 0. $$

### Theorem 4.1

Under the conditions of Lemma 2.2 and $|\lambda |< \frac{\alpha }{\max _{1\le i \le N}\sum _{j=0}^{N}h\eta _j|K_{ij}|},$ the solution *U* of (11) converges $\varepsilon$-uniformly to the solution *u* of (1). For the error of approximate solution the following bound holds

$$\begin{aligned} \Vert U-u\Vert \le Ch^2. \end{aligned}$$

### Proof

Applying the maximum principle, from (18) we have

$$\begin{aligned} \Vert z\Vert\le \alpha ^{-1}\Vert R-\lambda h\sum _{j=0}^{N}\eta _jK_{ij}z_j\Vert ,\\\le \alpha ^{-1}\Vert R\Vert +|\lambda |\alpha ^{-1}\max _{1\le i \le N}\sum _{j=0}^{N}h\eta _j|K_{ij}|\Vert z\Vert , \end{aligned}$$

hence

$$\begin{aligned} \Vert z\Vert \le \frac{\alpha ^{-1}\Vert R\Vert }{1-|\lambda |\alpha ^{-1}\max _{1\le i \le N}\sum _{j=0}^{N}h\eta _j|K_{ij}|} \end{aligned}$$

which implies of

19 $$\begin{aligned} \Vert z\Vert \le C\Vert R\Vert . \end{aligned}$$

Further we estimate for $\Vert R\Vert$. Thereby

$$\begin{aligned} \begin{aligned} \mid R\mid =\mid R_1 +R_2\mid=\left| -\varepsilon u_i''+\frac{\varepsilon }{\psi _{i}^2}\left( h^2u_i''+\frac{h^4}{12}u^{(iv)}(\xi )\right) + \frac{l}{180}h^4u^{(iv)}(\xi )\right| . \end{aligned} \end{aligned}$$

Using truncated Taylor series expansion of the denominator function $\frac{1}{\psi _{i}^2}= \frac{1}{h^2} - \frac{\zeta }{(12\varepsilon )} + \frac{\zeta ^{2}h^{2}}{(240\varepsilon ^{2})}$ [18] and this result into

$$\begin{aligned} \begin{aligned} \mid R\mid =&\left| (\frac{\varepsilon }{h^2} - \frac{\zeta }{12} + \frac{\zeta ^{2}h^{2}}{240\varepsilon })(h^2u_i''+\frac{h^4}{12}u^{(iv)}(\xi ))-\varepsilon u_i'' +\frac{l}{180}h^4u^{(iv)}(\xi )\right| \\ \le&\left|(\varepsilon - \frac{\zeta h^2}{12} + \frac{\zeta ^{2}h^{4}}{240\varepsilon })(u''(x_i)+\frac{h^2}{12}u^{(iv)}(\xi ))-\varepsilon u''(x_i)+\frac{l}{180}h^4u^{(iv)}(\xi )\right|\\ \le&\left|\left( -\frac{\varepsilon }{12}u^{(4)}(\xi )-\frac{\zeta }{12}u_i''\right) h^2+\left( \frac{\zeta ^2}{240\varepsilon }u_i''+\left( \frac{l}{180}-\frac{\zeta }{144}\right) u^{(4)}(\xi )\right) h^4 +\left( \frac{\zeta ^2}{2880\varepsilon }u^{(4)}(\xi )\right) h^6 \right|. \end{aligned} \end{aligned}$$

Using the bounds on the derivatives and Lemma 4.3 gives

$$\begin{aligned} \mid R\mid \le \left| \left( \frac{\varepsilon }{12}-\frac{\zeta }{12}\right) h^2+\left( \frac{l}{180}-\frac{\zeta }{144}+\frac{\zeta ^2}{240\varepsilon }\right) h^4+\frac{\zeta ^2}{2880\varepsilon }h^6\right| , \end{aligned}$$

using the relation $h^2>h^4> h^6 >\dots$ and for the case $O(h^4)\approx O(\varepsilon )$, we obtain

20 $$\begin{aligned} \Vert R\Vert \le Ch^2. \end{aligned}$$

The bound (20) together with (19) completes the proof. $\square$

## Numerical results and discussion

To verify the established theoretical results in this paper, we perform an experiment using the proposed numerical scheme on the problem of the form given in (1). We used the double mesh principle to estimate the maximum absolute error.

### Example 5.1

Consider the singularly perturbed problem from [3]

$$\begin{aligned} -\varepsilon u'' + u +\frac{1}{2}\int _{0}^{1}x\ u(s)ds=x-\varepsilon +\varepsilon e^{\frac{-x}{\varepsilon }},  u(0)=1, u(1)=2-\varepsilon +\varepsilon e^{\frac{-1}{\varepsilon }}. \end{aligned}$$

### Example 5.2

Consider singularly perturbed problem from [9]

$$\begin{aligned} -\varepsilon u'' +(2-e^{-x})u+\frac{1}{2}\int _{0}^{1}\left( e^{xcos(\pi s)}-1\right) u(s)ds=\frac{1}{1+x},  u(0)=1,  u(1)=0. \end{aligned}$$

### Example 5.3

Consider the particular problem

$$\begin{aligned} -\varepsilon u'' +(2-e^{-x})u+\frac{1}{2}\int _{0}^{1}\left( e^{xcos(\pi s)}-1\right) u(s)ds=x,  u(0)=1,  u(1)=2. \end{aligned}$$

The maximum absolute error is calculated using the formula $E_{\varepsilon }^{N}=\max _{0\le i\le N}|u^{N}(x_{i})-u^{2N}(x_{2i})|,$ and rate of convergence by formula $Roc^{N}=\log 2\left( \frac{E_{r}^{N}}{E_{r}^{2N}}\right)$.

**Fig. 1:**
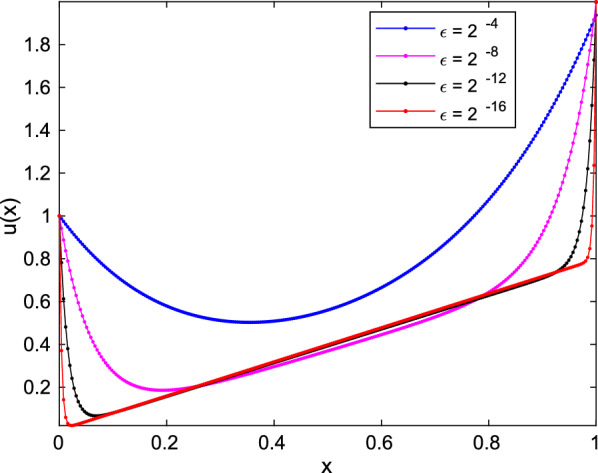
Solutions profile of Example 5.3 with the boundary layer formation as $\varepsilon$ goes small for $N=2^8$

**Table 1:** The maximum absolute error and rate of convergence of Example 5.1

$$ \varepsilon \downarrow $$	N $$ \rightarrow $$ $$ 2^5 $$	$$ 2^6 $$	$$2^7 $$	$$ 2^8 $$	$$ 2^9 $$	$$ 2^{10} $$
$2^{0}$	4.2307e-06	1.0587e-06	2.6468e-07	6.6170e-08	1.6505e-08	4.0694e-09
	1.9986	2.0000	2.0000	2.0033	2.0200	
$2^{-2}$	1.7772e-05	4.4479e-06	1.1124e-06	2.7811e-07	6.9524e-08	1.7294e-08
	1.9984	1.9994	1.9999	2.0001	2.0072	
$2^{-4}$	2.9647e-05	7.5264e-06	1.8872e-06	4.7238e-07	1.1812e-07	2.9529e-08
	1.9779	1.9957	1.9982	1.9997	2.0000	
$2^{-6}$	3.1288e-05	9.3513e-06	2.4711e-06	6.2516e-07	1.5690e-07	3.9252e-08
	1.7424	1.9200	1.9829	1.9944	1.9990	
$2^{-8}$	1.3865e-05	4.9014e-06	2.2887e-06	7.0094e-07	1.8356e-07	4.6429e-08
	1.5002	1.0987	1.7072	1.9330	1.9832	

**Table 2:** Comparison of the maximum absolute error of Example 5.1 of the proposed scheme and the result in [3]

$$ \varepsilon \downarrow $$	N $$ \rightarrow $$ $$ 2^5 $$	$$ 2^6 $$	$$2^7 $$	$$ 2^8 $$	$$ 2^9 $$	$$ 2^{10} $$
Proposed scheme						
$2^{0}$	4.2307e-06	1.0587e-06	2.6468e-07	6.6170e-08	1.6505e-08	4.0694e-09
$2^{-4}$	2.9647e-05	7.5264e-06	1.8872e-06	4.7238e-07	1.1812e-07	2.9529e-08
$2^{-8}$	1.3865e-05	4.9014e-06	2.2887e-06	7.0094e-07	1.8356e-07	4.6429e-08
$2^{-12}$	6.9416e-04	5.9134e-05	4.0396e-06	2.5841e-07	1.6151e-08	2.3062e-08
$2^{-16}$	4.0163e-03	1.2458e-03	1.8212e-04	1.5518e-05	1.0605e-06	6.7831e-08
Result in [3]						
$2^0$	0.00343868	0.00198874	0.00110332	0.00060368	0.00030394	0.00015197
$2^{-4}$	0.01032126	0.00605257	0.00338123	0.00185003	0.00094445	0.00047551
$2^{-8}$	0.01125894	0.00660244	0.00368841	0.0020181	0.00103025	0.00051871
$2^{-12}$	0.011200979	0.00656845	0.00366942	0.00200771	0.00102495	0.00051604
$$ 2^{-16}$$	0.0112049	0.00657075	0.00367071	0.00200842	0.00102531	0.00051622

**Table 3:** The maximum absolute error and rate of convergence of Example 5.2

$$ \varepsilon \downarrow $$	N $$ \rightarrow $$ $$ 2^5 $$	$$ 2^6 $$	$$2^7 $$	$$ 2^8 $$	$$ 2^9 $$	$$ 2^{10} $$
$2^{0}$	1.4021e-05	3.5078e-06	8.7725e-07	2.1932e-07	5.4820e-08	1.3692e-08
	1.9990	1.9995	2.0000	2.0003	2.0014	
$2^{-4}$	8.3355e-05	2.0870e-05	5.2203e-06	1.3052e-06	3.2632e-07	8.1577e-08
	1.9978	1.9992	1.9999	1.9999	2.0001	
$2^{-8}$	2.0102e-04	5.1032e-05	1.2800e-05	3.2036e-06	8.0104e-07	2.0027e-07
	1.9779	1.9953	1.9984	1.9997	1.9999	
$2^{-12}$	2.5085e-04	7.6354e-05	1.9909e-05	5.0336e-06	1.2619e-06	3.1570e-07
	1.7160	1.9393	1.9838	1.9960	1.9990	
$2^{-16}$	2.0675e-04	7.6047e-05	1.9640e-05	5.7644e-06	1.4992e-06	3.7847e-07
	1.4429	1.9531	1.7686	1.9430	1.9859	
$2^{-20}$	2.1327e-04	1.0804e-04	5.2959e-05	1.9586e-05	3.9418e-06	9.8186e-07
	1.9812	1.8286	1.4351	2.3129	2.0053	

**Table 4:** Comparison of the maximum absolute error of Example 5.1 of the proposed scheme and the result in Example 5.2

$\varepsilon \downarrow$	N $\rightarrow$ $2^5$	$2^6$	$2^7$	$2^8$	$2^9$	$2^{10}$
Proposed scheme						
$2^{0}$	1.4021e-05	3.5078e-06	8.7725e-07	2.1932e-07	5.4820e-08	1.3692e-08
$2^{-2}$	4.0894e-05	1.0233e-05	2.5588e-06	6.3973e-07	1.5993e-07	3.9962e-08
$2^{-4}$	8.3355e-05	2.0870e-05	5.2203e-06	1.3052e-06	3.2632e-07	8.1577e-08
$2^{-6}$	1.3785e-04	3.4622e-05	8.6671e-06	2.1673e-06	5.4188e-07	1.3547e-07
$2^{-8}$	2.0102e-04	5.1032e-05	1.2800e-05	3.2036e-06	8.0104e-07	2.0027e-07
Result in [9]						
$2^{0}$	0.02882363	0.00729132	0.00183933	0.00046239	0.00011608	0.00002906
$2^{-2}$	0.02860477	0.00725102	0.0018317	0.00046143	0.00015067	0.00003785
$2^{-4}$	0.04001304	0.01015697	0.00257469	0.00065085	0.0001643	0.00004139
$2^{-6}$	0.04331213	0.01100204	0.00279084	0.00070696	0.00017896	0.00004527
$2^{-8}$	0.04342876	0.01104697	0.00280418	0.00071231	0.00018094	0.00004593

**Table 5:** The maximum absolute error and rate of convergence of Example 5.3

$$ \varepsilon \downarrow $$	N $$ \rightarrow $$ $$ 2^5 $$	$$ 2^6 $$	$$2^7 $$	$$ 2^8 $$	$$ 2^9 $$	$$ 2^{10} $$
$2^{0}$	4.5386e-06	1.1365e-06	2.8421e-07	7.1060e-08	1.7782e-08	4.4008e-09
	1.9976	1.9996	1.9998	1.9986	2.0146	
$2^{-4}$	4.7504e-05	1.1867e-05	2.9661e-06	7.4148e-07	1.8537e-07	4.6342e-08
	2.0011	2.0003	2.0001	2.0000	2.0000	
$2^{-8}$	2.6857e-04	6.9095e-05	1.7495e-05	4.3794e-06	1.0957e-06	2.7394e-07
	1.9586	1.9816	1.9981	1.9989	1.9999	
$2^{-12}$	6.6845e-04	3.0691e-04	8.1741e-05	2.0780e-05	5.2171e-06	1.3072e-06
	1.1230	1.9087	1.9759	1.9939	1.9968	
$2^{-16}$	1.0737e-03	2.5600e-04	1.9205e-04	8.1349e-05	2.1584e-05	5.4813e-06
	2.0684	1.4466	1.2393	1.9142	1.9774	
$2^{-20}$	1.2196e-03	6.5715e-04	3.0150e-04	6.8690e-05	4.9737e-05	2.0647e-05
	1.9211	1.7241	2.1340	1.6578	1.6684	

The error analyses performed in this work reveal that the proposed method is second-order $\varepsilon$-uniformly convergent. This result, summarized in Theorem 4.1, is confirmed by numerical results displayed in Table 1, Table 3 and Table 5, where we computed the maximum absolute errors and rates of convergence for different values of mesh size *N* and perturbation parameter $\varepsilon$ for Examples 5.1, 5.2 and 5.3, respectively. On Table 1, Table 3 and Table 5, one can observe that, as $\varepsilon$ goes small the maximum absolute error of developed scheme becomes stable and bounded. This indicates that maximum absolute error of the scheme is independent of the perturbation parameter $\varepsilon$, implying that the scheme is $\varepsilon$-uniformly convergent. In Table 2, we compared the result of the proposed scheme with the results in [3] for Example 5.1 and in Table 4, we compared the result of the proposed scheme with the results in [9] for Example 5.2. As one can observes in these tables results in the proposed method is better than that exists in [3] and [9].

In order to show the physical behaviour of the given problem, we give plots of the computed solutions for different values of $\varepsilon$. In Fig. 1, the profile of the solution is given for different values of the perturbation parameter $\varepsilon$ with boundary layer formulation as $\varepsilon$ goes small.

## Conclusion

In this paper, a linear second-order singularly perturbed Fredholm integro-differential equation has been considered. This problem is solved numerically on a uniform mesh using a non-standard finite difference for the differential part and a composite Simpson’s 1/3 rule for the integral part. The stability and convergence analysis of the proposed scheme are proven. Three examples are used to investigate the applicability of the scheme. Effect of the perturbation parameter on the solution of the problem is shown using figures. It is demonstrated that the method is uniformly convergent (i.e., independent of the perturbation parameter), with a second order of convergence. Performance of the proposed scheme is investigated by comparing the results with those of prior studies. It has been found that the proposed method gives more accurate and stable results.

## Data Availability

The original contributions presented in the study are included in the article/supplementary materials, further inquiries can be directed to the corresponding author.
